# Serological Humoral Immunity Following Natural Infection of Children with High Burden Gastrointestinal Viruses

**DOI:** 10.3390/v13102033

**Published:** 2021-10-09

**Authors:** Mark R. Zweigart, Sylvia Becker-Dreps, Filemón Bucardo, Fredman González, Ralph S. Baric, Lisa C. Lindesmith

**Affiliations:** 1 Department of Epidemiology, University of North Carolina, Chapel Hill, NC 27599, USA; mrz@email.unc.edu (M.R.Z.); sbd@email.unc.edu (S.B.-D.); 2 Department of Family Medicine, University of North Carolina, Chapel Hill, NC 27599, USA; 3 Department of Microbiology, National Autonomous University of Nicaragua, León 21000, Nicaragua; fili_bucardo@med.unc.edu (F.B.); fredman.gonzalez@cm.unanleon.edu.ni (F.G.)

**Keywords:** rotavirus, norovirus, sapovirus, immunity, *Caliciviridae*, gastroenteritis, diarrhea, antibody, correlate of protection, histo-blood group antigen

## Abstract

Acute gastroenteritis (AGE) is a major cause of morbidity and mortality worldwide, resulting in an estimated 440,571 deaths of children under age 5 annually. Rotavirus, norovirus, and sapovirus are leading causes of childhood AGE. A successful rotavirus vaccine has reduced rotavirus hospitalizations by more than 50%. Using rotavirus as a guide, elucidating the determinants, breath, and duration of serological antibody immunity to AGE viruses, as well as host genetic factors that define susceptibility is essential for informing development of future vaccines and improving current vaccine candidates. Here, we summarize the current knowledge of disease burden and serological antibody immunity following natural infection to inform further vaccine development for these three high-burden viruses.

## 1. Introduction

**Figure 1:**
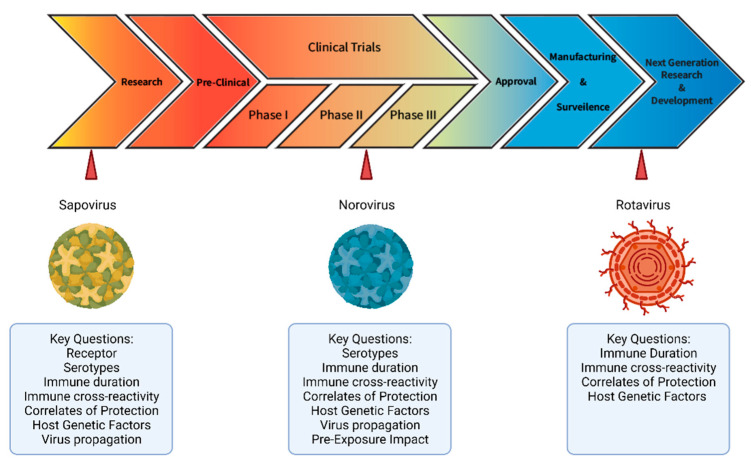
Graphical representation of current stages of research and development for sapovirus, norovirus, and rotavirus vaccines. Created with Adobe Illustrator and BioRender.com (accessed on 05 October 2021).

Rotavirus, norovirus, and sapovirus are nonenveloped RNA viruses that together cause a high proportion of the childhood gastroenteritis burden. In the multi-site Malnutrition and Enteric Disease Study (MAL-ED) birth cohort study, these three pathogens occupied three of the four top rankings of pathogens with the highest attributable incidence of diarrhea in children under 12 months of age [1]. A true success story in addressing the burden of childhood gastroenteritis has been the development and global roll-out of oral rotavirus vaccines. Despite somewhat lower vaccine effectiveness in low- and middle- income countries (LMIC), countries that added rotavirus vaccines to their national immunization schedules experienced a median reduction of 59% (IQR, 46–74) in rotavirus hospitalizations and a median reduction of 36% (IQR, 28–46) in all-cause gastroenteritis mortality [2]. While norovirus vaccines have reached phase II trials in children [3,4], to date, there are no licensed vaccines available against either of the high-burden caliciviruses, norovirus or sapovirus (Figure 1).

Due to their important contribution to gastroenteritis burden, elucidating the development of immunity to these three viruses is needed to understand patterns of susceptibility in populations and guide the development of future vaccines. Seminal birth cohort studies of rotavirus conducted in the 1990′s broadened our understanding of the development of natural immunity and guided rotavirus vaccine development [5]. Understanding protective immunity to rotavirus provides a template to guide future research on norovirus and sapovirus. However, a significant challenge to understanding immunity to caliciviruses is the inability to easily grow these viruses in cell culture to produce viral antigens and perform infectivity studies. Instead, for norovirus, adult human challenge studies have played a pivotal role in understanding immunity, but may not reflect immunity in naïve infants, who suffer the greatest burden of disease. A comprehensive understanding of sapovirus immunity remains in its infancy, representing a clear and present priority for the development of vaccines.

New data on host genetic susceptibility is further shaping our understanding of protection against rotavirus and norovirus. Histo-blood group antigens (HBGA) may mediate rotavirus and norovirus cellular attachment, and host HBGA phenotypes affect susceptibility to rotavirus and norovirus on a genotype-specific basis [6,7]. These host factors impact vaccine effectiveness which also help explain genotype distributions in populations [8,9].

The purpose of this review is to share recent knowledge and important gaps in the field on humoral immunity to rotavirus, norovirus, and sapovirus.

## 2. Rotavirus

### 2.1. Rotavirus Structure and Antigenic Characteristics

Rotavirus is an icosahedral triple-layered non-enveloped virus measuring 100 nm in diameter which contains 11 segments of double stranded RNA that encode six structural (VP1, VP2, VP3, VP4, VP6, and VP7) and six nonstructural proteins (NSP1 to NSP6) [10]. The middle layer is formed by VP6, a highly immunogenic protein, that elicits group-specific antibodies and defines serogroups (A to J) [11]. The outer layer is formed by VP7, which is also highly immunogenic and defines G (glycoprotein) genotypes within serogroups [12,13]. VP4 is a spike protein inserted into the VP7 protein, is highly immunogenic and defines the P (protease-sensitive) genotypes [12,13]. During infection, VP4 is cleaved into VP5* and VP8*; VP8* then functions as a ligand for cell attachment [14].

VP7 and VP4 are highly diverse genetically. To date, 41 G and 57 P genotypes have been recognized to infect human and animals by the Rotavirus Classification Working Group. However, only 4 G genotypes (G1, G2, G3 and G4) mainly in combination with 2 P genotypes (P[4] and P[8]) are globally prevalent in human populations, each of them showing fluctuations in incidence over time. The patterns of genotype variability over time suggests that genotype-specific immunity reduces transmission and disease burden, but sterilizing immunity is never fully achieved in most individuals. New G genotypes, such as G9 and G12 have emerged and become more common [15,16]. Moreover, animal-derived rotaviruses have emerged regionally (for example, porcine-like G5, bovine-like G8 and equine-like G3) and can spread globally [17,18,19,20,21,22,23,24,25,26,27]. The cross-species movement of animal derived rotavirus into humans may be due to co-infections with human and animal rotavirus strains, giving rise to reassortant strains which cannot be contained by herd immunity. A common observation is that animal-reassortant viruses retain the human P genotype, such as P[8].

### 2.2. Rotavirus Pathogenesis

Rotavirus infections cause non-bloody watery diarrhea, vomiting of short duration, and fever, and are associated with a limited inflammatory response [28,29]. Rotavirus infects the mature enterocytes in the mid and upper part of the villi of the small intestine [29]. Rotavirus-induced diarrhea is considered osmotic and non-inflammatory with diarrhea most likely due to extracellular accumulation of solutes and malabsorption as a result of enterocyte damage or death; or decreased epithelial absorptive function [29]. Another proposed mechanism is secretory diarrhea due to the effects of the viral enterotoxin, NSP4, on activation of the enteric nervous system. Rotavirus can exert effects on the central nervous system through nerve gut-brain communication, via the release of mediators, such as the rotavirus enterotoxin NSP4, which stimulates neighboring enterochromaffin cells in the intestine to release serotonin and activate both enteric neurons and vagal afferents to the brain [30]. There is evidence that the serotonin (5-HT3) receptor is involved in rotavirus-induced diarrhea by promoting intestinal motility [31] but not increased permeability [32]. There is also evidence of extra-intestinal rotavirus infection with antigenemia, and viremia commonly detected in hospitalized children within 10 days after onset of symptoms [33,34,35,36,37].

### 2.3. Mechanisms of Protection against Rotavirus Infection and Disease

There are two proposed mechanisms of protection against rotavirus infection. The first suggests that the protection is due to neutralizing antibodies that recognize serotype-specific epitopes on VP4 or VP7 proteins that block virus infection and entry [38]. The second proposes that protection is due to other effectors like non-neutralizing antibodies (Fc mediated is yet to be reported) and possibly T-cells (CD8 and/or CD4) [39,40,41]. Other effectors may include antibodies that recognize VP6, NSP4 or products of interferon-inducible genes like viperin [42,43,44,45,46,47]. For instance, immune responses against VP6, mediated by IgA, have been shown to inhibit viral replication in vitro and in vivo, and VP6 neutralization seems to be intracellular [45,46]. In addition, systemic and intestinal antibody responses to NSP4 enterotoxin have been documented in children and gnotobiotic pigs [42,43,44]. Regarding cellular protection, rotavirus T-cell studies in children have shown that rising antibody levels are accompanied by T-cell responses, but this cellular response is transient and disappears within one year [48,49,50]. These observations have led to the suggestion that rotavirus protection early in life is mediated by neutralizing antibodies and that serial infections may be needed for the development of stable memory T-cell populations [48]. Host genetics are another important factor in the protection against rotavirus. “Non-secretor“ individuals do not express the H type-1 histo-blood group antigen (HBGAs) on the intestinal epithelium, which seems to be required for cell attachment by the common rotavirus genotypes, P[8] and P[4] [51]. Several other factors, such as breastfeeding, intestinal microbiome composition, environmental enteropathy, and nutritional status have been suggested to either contribute to or impede immune protection against rotavirus acute gastroenteritis [52,53,54,55,56,57,58,59].

### 2.4. Host Genetics and Health Outcomes

HBGAs are a family of complex carbohydrates classified by terminal glycosylation patterns found in intestinal and other mucosa [6]. Innate susceptibility to rotavirus and human norovirus is regulated by polymorphisms in the genes that encode alpha 1,2 fructosyltransferase 2 (FUT2) (secretor enzyme) and Lewis enzyme (FUT3), and glycosyltransferases A and B [60,61,62,63]. These enzymes regulate glycosylation, starting at the precursor to H_1_. Secretor enzyme produces H_1_, which can be further modified by Lewis enzyme to form Lewis b (Leb). A and/or B antigens can be added to H_1_ in addition to Leb. Non-secretors (FUT2 negative) can only express a very limited assortment of HBGAs in mucosal secretions. Structural studies on the interaction between the rotavirus ligand VP8* and histo-blood group antigens (HBGAs) show a conserved binding site on P[14] that specifically interacts with A-type HBGAs, suggesting that rotavirus pathogenesis is influenced by genetically controlled expression of different HBGAs in human populations [64]. Building upon these early structural studies, a saliva binding study showed that the VP8* of the P[4] and P[8] genotypes bind to saliva containing the Lewis B and secretor HBGAs, while the P[6] genotype was shown to bind to the secretor antigen only. These observations were later replicated using synthetic HBGAs [65]. A more recent study provided structural evidence for the interactions of the P[8] genotype with the secretor HBGA precursor, but binding experiments did not support interactions with Lewis A or B HBGAs [7]. Further structural studies proposed the hypothesis that evolution of genogroup P[II] RV (P[8], P[4] and P[6]) progressed from animals to humans under the selection of type 1 HBGAs guided by stepwise host synthesis of type 1 ABH and Lewis HBGAs [66].These laboratory observations have been supported by epidemiological studies showing that P[8] and P[4] genotypes infect Lewis B secretor children preferentially and P[6] infects Lewis-negative children preferentially, irrespective of the secretor phenotype [6,67,68,69,70]. The rotavirus P[6] genotype is more prevalent in sub-Saharan African and South-East Asian countries than in other populations, coinciding with a higher proportion of Lewis-negative phenotypes, reaching up to 30% of this population [60,68,71,72]. P[6] is also associated with neonatal infection and the structural basis for the age-restricted tropism have been reported for some P[6] strains. Authors suggest that this finding may be explained by the abundance of regulated unbranched glycans in the neonatal gut, that decline over time [73]. P[6] rotavirus is also found to circulate in pigs and there is evidence of interspecies transmission [74,75,76,77,78]. Human and porcine HBGAs share homology and type-A and H type 1 HBGAs are the most prevalent in pigs [79,80]. These similarities could form the basis for a mechanism of zoonotic and interspecies transmission of rotaviruses [79]. Studies examining the association between antibody response and HBGAs profiles have shown significantly higher IgG, IgA, and neutralizing antibody titers to rotavirus in secretor adults as compared to non-secretors [81]. Moreover, following rotavirus vaccination of children, IgA response and vaccine strain shedding are associated with secretor status [9,80,82,83,84].

### 2.5. Correlates of Rotavirus Immunity and Protection in Children

A central goal of vaccine research and the study of natural history of viral infections is to identify vaccine induced or naturally induced immune responses that predict protection from infection or disease, (Table 1). Among the predictors of protection from rotavirus infection and disease the most recognized are serum rotavirus IgA (>1:800) and VP7 specific IgA (>1:200) [85,86], followed by serum rotavirus IgG (>1:6400) and VP7 specific IgG (>1:800), respectively [86]. Fecal IgA, antibody secreting cell (ASC), rotavirus CD4+, rotavirus specific or neutralizing antibodies that recognize NSP4, VP6, VP4, VP7, and also epitope-specific neutralizing antibodies to VP7 have been also studied [13,87]. Sterilizing immunity could not be induced by natural rotavirus infection or vaccination due to antigenic drift and shift caused by the error-prone viral RNA polymerase, and also due to other evolutionary characteristics of rotavirus like gene reassortment, gene recombination and interspecies transmission [88]. Historic studies in a gnotobiotic pig model of rotavirus infections have shown that protective immunity against diarrhea depends on the production of IgA and IgG antibody-secreting cells and memory B cell responses at the site of viral replication in the ileum; IgA memory B-cells decline substantially in about 3 months after infection [89]. Studies of natural rotavirus infections in children showed a similar positive association between IgA and IgG antibodies and protective immunity against rotavirus infection and diarrhea, but after adjusting for age, the effect was only partial [90]. Following immunization, anti-rotavirus IgA Geometric Mean Concentration (GMC) was associated with a decline in vaccine efficacy. Efficacy during the first 2 years of life was significantly lower in countries with vaccine-elicited IgA GMC < 90 (44%; 95% confidence interval [CI], 30–55) compared to countries with GMC > 90 (85%; 95% CI, 82–88) [91]. NSP4-specific antibodies may be important in protecting against clinical symptoms of rotavirus infection [42,43,92]. In children with rotavirus gastroenteritis and in animal models, NSP4 induces humoral immune responses with modest IgG seroconversion rates (54–70%) and IgA being barely or not detected at all [42,44,93,94,95]. The titers of NSP4 specific IgG antibody have been found to be transient, increasing with age, peaking between 12–23 months of age and dropping to minimal levels afterwards [96]. A significant proportion of children who did not develop diarrhea associated with rotavirus infection had antibodies to NSP4 in acute-phase serum [44]. There is evidence that humans can circumvent the extensive serotype diversity of circulating rotavirus strains by generating frequent heterotypic neutralizing antibody responses to VP7, VP8*, and most often, to VP5* after natural infection [97]. Further research is warranted to understand whether VP6 IgG contributes to protection [37].

### 2.6. Impact of Rotavirus Vaccination on Disease Burden in Children

Current rotavirus vaccines have been available since 2006 and the World Health Organization (WHO) recommended universal infant rotavirus vaccination in 2009 [2]. As of 2020, about 70%, 60% and 45% of the African, American, and European countries have introduced these vaccines in their national immunization schedules. Before vaccine introduction, it was estimated that rotavirus accounted for 527,000 deaths annually or 29% of all deaths due to diarrhea among children <5 years of age, with the majority of the fatal cases occurring in LMIC countries [98]. In contrast, in 2016, the fatalities due to rotavirus decreased to 128,500 among children <5 years throughout the world, with 104,733 deaths occurring in sub-Saharan Africa [99]. Data from the WHO-coordinated Global Rotavirus Surveillance Network reported a 40% relative decline in rotavirus positive specimens among children <5 years old hospitalized for diarrhea [100]. Among children <5 years old, there was a median reduction of 59% (IQR, 46–74) in rotavirus hospitalizations, 36% (IQR, 23–47) in acute gastroenteritis hospitalizations, and 36% (IQR, 28–46) in mortality due to all-cause acute gastroenteritis [2]. Current and new licensed rotavirus vaccines, contain live attenuated strains that are given orally to children very early in life to induce either heterotypic or homotypic responses [101]. RotaTeq (G1–4/P[8]) and Rotasiil (G1–4 and G9) contain reassortant strains with a bovine rotavirus backbone and VP4 and VP7 from human origin, based on the concept of genotype-specific protection. In contrast, Rotarix (G1P[8]) and Rotavac (G9P[11]) consist of attenuated human RV strains and are based on the concept that one genotype induces heterotypic protection [102].

### 2.7. Conclusions and Next Steps

Globally prevalent and emerging rotavirus strains retain the ligand VP4 specificity (P[8] and P[4]) while changing the outer layer of VP7 (G1–4, G9, G12). The introduction of rotavirus vaccines against severe gastroenteritis has proven to be highly effective, more likely due to induction of mucosal IgA responses. Emerging observations suggest that immune response to rotavirus vaccines is dependent on host genetic factors such as the Lewis and secretor phenotypes. Efficacy studies examining these factors may inform the improvement of rotavirus vaccines. Vaccine developers might improve their vaccine design based on what is learned from observational and mechanistic studies on host susceptibility.

## 3. Norovirus

### 3.1. Norovirus Burden of Disease

Human norovirus is the leading cause of acute viral gastroenteritis, potentially accounting for up to 1/5th of all gastroenteritis cases worldwide. More than 200,000 children per year die from complications of human norovirus infection [103]. Children between 6 months and 5 years of age are at greatest risk of death [104]. Norovirus disease burden is similar to that of rotavirus before the development and implementation of rotavirus vaccines [2]. The economic costs of norovirus in the United States alone is estimated to exceed 10 billion dollars per year [105]. Symptoms common among all age groups include diarrhea, vomiting, nausea, and stomach pain; however, children under 5 years and adults over 65 years are at greater risk of severe disease [106,107,108]. Symptoms begin 12–48 h after initial exposure with a recovery within 2–5 days in acute cases in adult immune-competent individuals [109]. Infection may persist for longer in young children [110]. Secretor positive children with norovirus AGE present more severe symptoms than children with non-norovirus AGE [111,112,113,114]. Vomiting is a primary symptom in children seeking medical care for human norovirus AGE [115], sometimes with intensities indistinguishable from rotavirus infections.

### 3.2. Virus Background

Human norovirus is a calicivirus, with 3 open reading frames on a positive sense RNA genome of approximately 7.5 kilobases. ORF 1 encodes the viral replication machinery. ORFs 2 and 3 encode the major (VP1) and minor (VP2) capsid proteins. The viral capsid is composed of 90 dimers of the major capsid protein. The capsid protein is divided into functional domains; the interior shell and protruding domain that extends away from the shell surface [116]. Ligand binding and strain primary antigenic determinant for human norovirus comprise the most-exterior region of the protruding domain, the P2 subdomain [117,118,119]. Human norovirus is genetically and antigenically diverse. To date, there are 10 genogroups (G), with GI and GII being the primary causes of disease in humans [120,121]. GI is comprised of 9 genotypes and GII is comprised of 27 genotypes [121]. GII strains account for ~90% of infections and occur earlier in life than GI infections [122,123]. Infection follows a fecal-oral route, with infection possible with small amounts of virion exposure [124,125,126,127]. Once infected, children may shed virus for weeks post-infection [128]. Human norovirus is highly transmissible, especially in enclosed locations with a high density of people [103,129]. Highest mortality rates are recorded in long-term care/retirement faculties for adults and LMIC for children [130,131].

Little is known about in vivo replication of human norovirus, beyond infection of enterocytes in the small intestine. In vitro, infection of intestinal epithelial cells is facilitated by bile and ceramide [132]. Human norovirus capsid domains bind to select HBGAs in strain-specific patterns, determining genetic susceptibility of infection [60,61,62]. GII.4 variants are characterized by broad HBGA binding patterns and this breadth of ligand binding contributes to the global dominance of these stains [133,134]. Secretor-negative individuals are mostly protected from some genotypes that require H or AB antigens, such as GI.1 and GII.4 strains [8,60,61,62]. Exceptions to innate susceptibility for secretor-negative people have been documented for GI.2, GI.3, GII.1, GII.2, GII.3, GII.6, GII.7, GII.17, and rarely GII.4 [60,135,136], indicating genetic resistance is highly protective against human norovirus genotypes but does not provide complete protection from infection from all genotypes.

Young children are most at risk of infection and severe disease. Peak infection rates occur during the first two years of life with studies reporting >30 infecting genotypes in children under 18 years old and 22 genotypes in children ≤5 years old [122,123,137]. Globally, GII strains account for >90% of infections [122,123]. Within GII, GII.4 variants are responsible for 40->95% of infections in children [107,137,138]. GII.4 infections are most frequently the first infecting strain and are more likely to have severe symptoms than non-GII.4 infections [107,112,113,139].

Non-GII.4 genotypes, peak and ebb in prevalence [113,123,140]. GII.3, GII.6, GII.2, GII.12 and GII.17 are frequently detected [123,141,142,143]. GI.3 is the most frequently detected GI genotype [122,123]. Infecting strains are classified by the capsid sequence. Capsid genes commonly circulate with interchangeable polymerase types, as recombinant viruses [144]. The difference in virulence between recombinant viruses compared to non-recombinant viruses is under investigation but hampered by the lack of tools for evaluation [141,145,146,147,148]. Co-infection with other AGE viruses, bacteria and parasites are also common and understudied [137,149,150,151].

### 3.3. Correlates of Protection

Antigenicity and immunogenicity studies have focused on the major capsid protein as the primary target of antibody responses, although antibodies to non-structural proteins have been identified [152]. Serological evidence of human norovirus exposure is ubiquitous in children across income brackets. Both reactive IgG and antibody that blocks ligand binding in a surrogate neutralization assay (blockade antibody) responses have been detected in the sera of children <6 months of age. Antibodies present before 6 months of age are likely maternally derived and provide some degree of protection from infection in early infancy [104,153,154,155,156]. By 12 months of age, child-derived serum antibody titers are present [104,155,157]. By age 3, most children have experienced primary human norovirus infection, and seropositivity may reach >90% in some populations [48,153,157,158]. Generally, GI antibodies are less frequently detected and are detected at an older age than GII antibodies [48,156,157,159]. Serum antibodies to GII.4 strains are the most detected antibodies, aligning with global strain circulation patterns [160]. Virus exposure and antibody titers remain high into older adulthood [106,161,162].

Characterizing human norovirus serotypes would provide key information for designing rational vaccine platforms that provide cross-protection and for determining correlates of protection for bridging vaccine studies. Studies indicate there is little cross-genogroup protection in humans [154,160,163,164,165]. Studies have found few examples of repeat genotype infections but multiple infections within a genogroup are common, suggesting some genotypes may correspond to serotypes, at least in young children with limited exposure histories [165,166,167,168,169].

Surveying for reactive antibody is useful for prevalence studies but extrapolation of findings for human norovirus is complex. Increases in homotypic serum antibody titers is highly correlated with detection of viral strains in stool and determining infection [61,62,170]. DNA sequencing of PCR products generated from fecal extracted RNA can accurately determine an infecting viral strain but the extensive and expanding diversity of human norovirus genotypes limits the ability to screen sera for reactivity to matched VLP in many cases. Since infection and vaccination induce antibodies to non-neutralizing epitopes conserved within and across GI and GII genotypes [164,171,172] interpretation of antibody binding data alone is not definitive for determining genotype infection.

Antibodies induced by infection are likely protective. Children with GII.4 antibodies are less likely to get a GII.4 infection than children without GII.4 antibodies [153,173]. Treatment of infection in immunocompromised individuals with oral immunoglobulin preparations reduced symptoms/infection in some cases but not others, suggesting strain-matched lots of immunoglobulin may be needed for efficacy [174,175,176,177,178]. Little is known about functions of different antibody isotypes in protection from infection or about antibodies to viral components outside the major capsid protein. In adults, salivary and serum IgA, but not serum IgG, correlate with protection from infection/symptoms [61,179,180].

Antibody able to blockade VLP binding to carbohydrate ligand is the most often demonstrated correlate of protection and is applied widely in evaluating infection and vaccine responses [179,181,182,183,184]. The “blockade assay”, is a surrogate assay for measuring virus-neutralizing antibody that disrupts virus cellular docking mechanisms and primarily target epitopes in the surface-exposed P2 domain [119,164,171,185]. All tested blockade antibodies also neutralize virus in an in vitro cell culture model, validating the surrogate assay [171,172,186,187]. Unlike reactive antibodies, blockade antibodies are highly genotype specific [164,169,171,187], and readily discriminate between variants of GII.4 strains [164,171,188,189]. This high specificity contributes to the usefulness of the blockade assay as a correlate of protection. Elevated pre-challenge titers of blockade antibody correlate with reduced infection and/or symptoms post-infection in controlled human challenge studies in adults [179,182,190,191]. Reconstitution of variant-specific blockade antibody correlated with resolution of diarrhea in an immune compromised patient [192]. IgA and IgG serum antibodies and recombinant monoclonal antibodies have blockade potency [164,172,178,187,193,194,195]. The relationship between these serum antibodies and mucosal antibodies is under explored, but serum IgA, salivary IgA and blockade antibody titer correlate with each other [195,196]. One study, demonstrated IgA and IgG isolated from saliva had blockade activity, supporting common functionality between antibodies of the systemic and mucosal compartments [195]. Future studies focusing on mucosal immune compartments may strengthen the association of blockade antibody and protection and further define serotypes of human noroviruses.

Few investigations of blockade antibody have been described for children, identifying a critical knowledge gap needed to inform vaccine development and evaluation. From limited studies of children <6 months of age, maternal antibody includes blockade antibodies [104] and infection induces development of strain-specific blockade antibodies accompanied with increased serum antibody avidity [104,169]. In two studies of children less than 2 years old infected with GII.4.2009 New Orleans, blockade antibodies to GII.4.2009 New Orleans were low in acute sera and increased >4-fold in convalescent samples, indicating very young children generate potentially protective antibody responses to infection [173,197]. Blockade antibody cross-reacted, although to a lower titer, with the closely related variant GII.4.2012 Sydney but not the distantly related variant GII.4.1999 US95/96, supporting findings with adult sera demonstrating antibody-mediated immune escape by evolved GII.4 variants [133,198,199].

Although infected and vaccinated adults have cross-genotype blockade antibody responses that persist for at least one month [163,181,193,200], no cross-genotype blockade antibody responses have yet been characterized in children. It is unclear how many infections are needed to develop blockade antibody titer, what titer is needed to provide protection and how long these titers persist after infection. These questions would best be answered through birth cohort studies with continuous collection of stool and sera accompanied by RT-qPCR monitoring of both symptomatic and asymptomatic human norovirus infection. Blockade antibody assays with infection-matched virus-like particles as antigens would allow for precise measurement of development and duration of immunity and evaluation of cross-protection, key information for designing effective vaccines and metrics for measuring vaccine outcomes.

### 3.4. Vaccine Development

Two human norovirus vaccine candidates are in clinical trials in heathy adults. Both systems use VP1 based VLPs as immunogen. The Vaxart vaccine platform uses a VP1-expressing adenovirus vector along with a double stranded RNA adjuvant that delivers the immunogen at the gut mucosa via an oral tablet [183]. In a preliminary two dose study, a monovalent GI.1 vaccine was both safe and immunogenic and induced GI.1-specific blockade antibody titers two-fold in 61% of low dose participants and 78% of high dose participants at day 28 [183]. Further, VP1-specific IgA memory B cells and plasmablasts, and IgA+ plasmablasts expressing mucosal homing receptors were elevated at seven days following a single dose of vaccine [183]. Follow up study of a bivalent GI.I/GII.4 vaccine candidate using the same platform is currently in phase 1B trials (NCT03897309). Challenge studies to test the efficacy of either Vaxart vaccine candidate have yet to be conducted. The Takeda bivalent VLP vaccine comprises an intramuscular injection of purified VLP from VP1 of both GI.1 and GII.4 with Al(OH)_3_ adjuvant [181,191,201]. Immunization resulted in rapid, broad blockade antibody responses to the vaccine components, other GI and GII strains and two GII.4 strains not widely circulating, providing the first piece of evidence that vaccination may provide cross-protection across genotypes and GII.4 variants [181]. A 15/50 µg ratio of GI.1 to GII.4 VLP dose resulted in a balanced response to both VLP antigens [202]. Pan-immunoglobulin, IgA, and HBGA blockade antibodies remained elevated at day 365 following one dose [203]. Most importantly, the bivalent VLP vaccine demonstrated 68% efficacy for moderate/severe norovirus acute gastroenteritis caused by any human norovirus in a field study [201]. Immunity at the mucosal site of infection likely determines the outcome of virus challenge. However, correlates of protection at the gut mucosa are unknown, complicating predicting the success of any human norovirus vaccine.

There are significant challenges to the development of a human norovirus vaccine, primarily centered around the antigenic diversity among strains infecting young children. Globally, for the past 30 years, GII.4 variants have been the leading cause of human norovirus infection, in all age groups. Success of these viruses is facilitated by binding to a broad selection of HBGAs resulting in large susceptible populations and antigenic drift in neutralizing epitopes resulting in escape from herd immunity [119,133,189,204]. GII.4 evolution is the key obstacle to vaccination. To counter antigenic diversity and viral evolution, a multivalent immunogen platform is most likely to provide antibody breadth and protection, as antibodies to epitopes conserved across genogroups or genotypes provide little ligand blockade or neutralizing potential [205,206]. The human norovirus vaccine clinical trials have exclusively studied adults and antibody responses were predominantly memory recall responses [171,181,202,207,208]. Children are the primary target population for human norovirus vaccination, since they bear the highest burden of norovirus disease and vaccination of children would decrease disease in older adults as well [209]. Vaccines in the very young will have to elicit breadth across antigenically diverse viruses. This goal will potentially be confounded by limited exposure histories in infants. How the current vaccine candidates will perform under these conditions is unknown as is whether the mucosal/intramuscular immunogen delivery effects immune responses or protection. These questions should be a high priority for study.

## 4. Sapovirus

### 4.1. Burden of Disease across the Ages and State of Vaccine Development

Sapovirus is increasingly recognized as an important cause of childhood diarrhea. The Malnutrition and Enteric Disease (MAL-ED) study, a multi-site birth cohort study of enteric infections in LMICs, found sapovirus to have the second highest attributable incidence for diarrhea among all enteric pathogens studied in children under 24 months of age [1].

Sharing clinical characteristics with closely-related noroviruses, common symptoms of sapovirus gastroenteritis include vomiting and diarrhea [210]. Farkas, et al. reported the seroprevalence of sapovirus (Mex340 strain) in children between 20 and 24 months of age involved in a birth cohort was greater than 90%, showing the high incidence of infections in early childhood [211]. Also in young children, one study, found sapovirus to be s associated with lower cognitive scores [212]. In children in the US, sapovirus has been detected in 10% of cases seeking care for diarrhea [213].

Sapovirus is also responsible for gastroenteritis outbreaks including in nursing homes, highlighting, its contribution to the burden of gastroenteritis in older adults [214]. Finally, while sapovirus is primarily associated with acute gastroenteritis, it has also been detected in cases of chronic diarrhea among individuals with immunocompromising conditions [215,216,217]. Despite its importance to human health across the age spectrum, there have been little efforts made to develop vaccines against sapovirus.

### 4.2. Virus Structure and Genetic Diversity

Sapoviruses are non-enveloped, single-stranded, positive-sense RNA viruses. They form the genus sapovirus within the family *Caliciviridae*. The sapovirus genome consists of two open reading frames: ORF1 encodes the virus nonstructural proteins and major capsid protein, VP1, and ORF2 encodes VP2, the minor structural protein [218]. Sapoviruses have been divided into 19 genogroups, with viruses from four of these (GI, GII, GIV, and GV), including 18 genotypes are known to infect humans [219]. Of these genotypes, sapovirus GI.1 is the most frequently detected worldwide [220]. The cellular binding site of sapovirus is not known; unlike noroviruses, there is no evidence for an association between host HBGA expression and disease risk [221]. Until very recently, sapovirus could not be replicated in cell culture, presenting challenges for understanding immunity including limiting infectivity studies and the production of antigen needed to measure humoral immunity [222]. Recent advances in cell culture of sapovirus [222] provide optimism that a deeper understanding of sapovirus immunity may be possible in the near future once these methods have been further established.

### 4.3. Health Outcomes/Host Population

Sharing clinical disease characteristics with closely-related noroviruses, common symptoms of sapovirus gastroenteritis include vomiting and diarrhea, which typically resolve within one week [210]. A MAL-ED study of enteric infections in children in LMICs, found sapovirus to have the third highest attributable incidence for diarrhea of all enteric pathogens among children under 12 months of age, and the second highest attributable incidence among children between 12 and 24 months of age [1]. Further, a Bangladeshi study identified sapovirus as one of only two enteric pathogens associated with lower cognitive scores in children at 24 months of age [212]. In the US, sapovirus has also been detected in 10% of children under age 18 receiving care for diarrhea in both the inpatient and outpatient settings [213]. Sapovirus is also responsible for gastroenteritis outbreaks, including in day care centers [223,224], schools [223,225,226], hospitals, [227,228] and nursing homes [214,223,229,230]. The fact that sapovirus was identified as the second most common cause of gastroenteritis outbreaks in nursing homes in the UK highlights its contribution to the burden of gastroenteritis in older adults [214]. Finally, while sapovirus is primarily associated with acute gastroenteritis, it has also been detected in cases of chronic diarrhea among individuals with immunocompromising conditions [215,216].

### 4.4. Immune Protection against Sapovirus

Epidemiological data from the MAL-ED birth cohort show a modest reduction in the hazard of future sapovirus infections after a first infection [HR = 0.92 (92% CI, 0.82, 1.03)], which decreases further after 2 or more infections [HR = 0.78 (95% CI, 0.69, 0.88)] [231]. The low protection following infection may reflect the genetic diversity within this viral genus. Little is currently known about determinants of humoral immunity to sapovirus and correlates of protection. Humoral immunity to sapovirus in acute and convalescent samples was first demonstrated by Chiba, et al., in 1979 using purified viruses from infected stool samples [232]. Early seroprevalence studies showed that seroprevalence increases with age, reaching the highest levels in late childhood that continues into adulthood, and falls in adults over 60 years of age [233]. This suggests that early life infections may provide durable humoral immunity that lasts until immunosenescence occurs in old age, and burden again increases [214,233]. Information on protection afforded by humoral immunity to sapovirus includes a study by Nakata, et al., who analyzed 41 paired sera from infants involved in a sapovirus outbreak using radio-immunoassays [233,234]. Among the 23 infants without preexisting antibody, 73% developed clinical symptoms and showed increases in sapovirus-specific antibody titers. Among the 18 infants with preexisting antibody, only 17% became sick and all but 3 experienced increases in sapovirus-specific antibody titers. These data show that serum antibody correlated with protection against clinical disease and that boosting occurs upon re-infection. However, it is not known if serum antibody is the most important correlate of protection against sapovirus or whether it simply mirrors the antibody levels on the intestinal mucosa. As the small intestinal mucosa is the location of infection, it is presumed that fecal IgA antibodies are important to immune protection and that innate immune activation against viral infection is mediated through the interferon pathway [235]. Chronic sapovirus infections in individuals with immunodeficiencies show that clearance of infections depends on intact host immune responses [216,236].

There is also little known about the breadth of humoral immunity following sapovirus infection. Studies using baculovirus-expressed viral capsid proteins and hyperimmune antisera showed that sapoviruses differ antigenically from GI and GII noroviruses [237]. Studies with hyperimmune sera show that human sapovirus genogroups are antigenically distinct, as there is strong reactivity against VLPs of homologous sapoviruses, but little cross reactivity between genogroups [238]. Epidemiological studies provide additional insights on the breath of sapovirus immunity. In a Peruvian birth cohort it was found that repeat sapovirus infections do occur, but repeat infections with the same genotype are rare: over two years of surveillance; 59 of 82 children who experienced a sapovirus infection went on to have a repeat sapovirus infection, however only three repeat infections were of the same genotype [239,240]. These data suggest that first sapovirus infections provide immunity against homologous infections, but little protection against infections of different genogroups, and possibly different genotypes. Further, the low incidence of symptomatic sapovirus episodes in the first months of life in epidemiological studies supports early protection from maternal immunity [210,239].

### 4.5. Early Knowledge about Viral Epitopes

Little is known about the viral structure and antigenic characteristics of sapovirus. Other caliciviruses have a conserved domain of VP1 capsid, while the structures and sequences of the P domain are variable. To date, structural approaches to characterize sapovirus have used chimeric VP1 capsids from vesivirus, a genus in the *Caliciviridae* family; these investigations helped to predict the S and P domains and have shown that elevated P-dimers could expose immunoreactive epitopes [241]. Unfortunately, to date there are no high resolution VLP structures that allow a detailed structural analysis of the sapovirus particle [242].

The use of immunoinformatic tools can help to uncover the antigenicity of sapovirus, Amin et al. predicted the 3D structure of the capsid protein of human sapovirus using a homology model; they were able to predict five conserved epitopes for T-cells that may also have binding affinity for B -cells [243]. However, the prediction was based on an atomic structure of a native calicivirus of the genus vesivirus that showed only 27% identity and 42% similarity with the target sapovirus sequence, so caution is warranted [243]. A better understanding of antigenic properties and identification of immunogenic epitopes would inform future vaccine development.

### 4.6. What We Need to Move Forward with Better Understanding of Immunity

Much can be learned from the fields of rotavirus and norovirus to advance our understanding of humoral immunity and correlates of protection against sapovirus. Information on natural boosting, re-infection, and antibody persistence in children is limited. Also, the role of animal sapovirus strains in causing clinical disease or immune boosting has not been established. There is optimism for the future success of vaccines due to the predominance of a single genotype, [244,245,246] lack of epidemic strains (such as norovirus GII.4), and evidence for durable immunity through adulthood. Finally, new tools are emerging to facilitate these investigations, including the use of VLPs for antigen production and recent developments in cell culture propagation techniques [222].

## 5. Conclusions and Key Questions Moving Forward

Acute gastroenteritis caused by viruses is one of the major causes of death worldwide. Effective vaccines coupled with other effective preventive measures (improved water quality and sanitation, breastfeeding and nutritional interventions) are needed to relieve this burden of illness on vulnerable populations, primarily young children. Duration and breadth of immunity provided by infection and vaccination and how these outcomes are impacted by pre-exposure history and host genetics are key questions of concern (Figure 1). Study of birth cohorts should be prioritized to answer these questions. These studies would also yield valuable virus challenge inoculum for additional controlled human challenge models for vaccine and therapeutics evaluation [170,190]. New tools for norovirus and sapovirus reagent development and the pathways paved through prior research on rotavirus and norovirus humoral immunity, will aid investigators to more quickly answer these questions and others to guide vaccine development, including number of doses, which antigens to choose and whether booster doses will be necessary.

**Table 1:** Serological Correlates of Protection.

Virus	Correlate	Infection	Symptoms	Reference
Rotavirus	Rotavirus IgA and IgG in serum	X	X	[86,91,247]
	Genotype specific IgA and IgG in serum	X	X	[85]
	Rotavirus IgA in stools	X	X	[248,249]
	VP6 serum and fecal IgA	X		[45,46,250]
	NSP4 IgG		X	[42,93,94]
Human Norovirus	Salivary IgA	X	X	[61,180]
	Memory IgG cells		X	[39,89]
	Blockade Ab titer	X	X	[173,179,182,190]
	Hemagglutination Inhibition titer	X	X	[251]
	Serum IgA	X	X	[179]
Sapovirus	Serum Ig		X	[234]
